# Non‐Enzymatic RNA Backbone Proofreading through Energy‐Dissipative Recycling

**DOI:** 10.1002/anie.201703169

**Published:** 2017-05-03

**Authors:** Angelica Mariani, John D. Sutherland

**Affiliations:** ^1^ PNAC MRC Laboratory of Molecular Biology Francis Crick Avenue, Cambridge Biomedical Campus Cambridge CB2 0QH UK

**Keywords:** energy-dissipative processes, molecular evolution, nucleic acids, origin of life, RNA

## Abstract

Non‐enzymatic oligomerization of activated ribonucleotides leads to ribonucleic acids that contain a mixture of 2′,5′‐ and 3′,5′‐linkages, and overcoming this backbone heterogeneity has long been considered a major limitation to the prebiotic emergence of RNA. Herein, we demonstrate non‐enzymatic chemistry that progressively converts 2′,5′‐linkages into 3′,5′‐linkages through iterative degradation and repair. The energetic costs of this proofreading are met by the hydrolytic turnover of a phosphate activating agent and an acylating agent. With multiple rounds of this energy‐dissipative recycling, we show that all‐3′,5′‐linked duplex RNA can emerge from a backbone heterogeneous mixture, thereby delineating a route that could have driven RNA evolution on the early earth.

The prebiotic emergence of the first RNA oligomers is at the crux of the origin of life. Recent reports describe the possible abiotic production of monomeric RNA precursors from simple feedstocks,^1^, ^2^, ^3^, ^4^ but advancing to the next level of complexity has been hindered by several constraints.^5^, ^6^, ^7^ Foremost of these is the inherent lack of regiocontrol in the oligomerization of activated ribonucleotides. In the absence of complex macromolecular catalysts, new internucleotide linkages are inevitably forged as both 3′,5′‐ and 2′,5′‐phosphodiester bonds.^6^, ^7^, ^8^ The problem has prompted extensive investigation, ranging from synthetic efforts to favor natural 3′,5′‐bonds,^8^, ^9^, ^10^, ^11^ to the finding that, below a threshold level, backbone heterogeneity might be compatible with the catalytic and recognition properties of RNA.^12^ Nevertheless, the question of how RNA might have evolved to the exclusively 3′,5′‐linked material that is primarily employed by extant biology remains largely unanswered. The well‐known preferential hydrolysis of 2′,5′‐bonds in a helical context^11^, ^13^ might have played a key role in this transition, with 3′,5′‐linkages becoming enriched simply through the depletion of 2′,5′‐linked material. However, rather than just degrading RNA, a more plausible scenario would embrace recycling, with formation of new 3′,5′‐bonds through repair of the broken linkages. A theoretical model addressing this was proposed in 1977 by Usher,^14^ who envisioned that day/night alternation on the early earth could have created suitable conditions for the degradation of 2′,5′‐bonds and subsequent joining of the resulting fragments. However, Usher invoked dry‐state non‐templated oligomerization for the joining chemistry, and this is known to only slightly favor the formation of natural linkages^15^ and is limited by unfavorable equilibrium considerations to producing short fragments.^16^ Furthermore, hydrolysis followed by non‐templated synthesis would not allow the propagation of sequence information and, accordingly, we sought a scheme whereby 2′,5′‐bonds could be “corrected” to 3′,5′‐bonds through proofreading with retention of sequence. We reasoned that any such repair process would have an energetic cost, and hence sought an energy‐dissipative cycle^17^, ^18^ that would combine selective hydrolysis of duplex 2′,5′‐linkages with templated 3′,5′‐selective ligation chemistry.^19^

We recently reported that templated ligation of mixtures of short oligonucleotides terminating with 2′‐ and 3′‐monophosphates can be made 3′,5′‐selective by means of sequential acetylation and ligation chemistry. The selectivity derives from preferential 2′‐O‐acetylation of 3′‐monophosphate‐terminated oligomers, which can then undergo regiospecific ligation. Subsequent hydrolytic removal of the 2′‐O‐acetyl groups at the newly formed 3′,5′‐internucleotide linkages completes the process. In contrast, 2′‐monophosphate‐terminated oligomers within the starting mixture are inefficiently 3′‐O‐acetylated and instead cyclize to 2′,3′‐cyclic phosphates, which undergo slower ligation.^8^ We realized that if the selective hydrolysis of 2′,5′‐phosphodiester bonds could be coupled to this ligation chemistry, a new model would emerge in which each cycle of hydrolysis and synthesis would convert 2′,5′‐linkages into 3′,5′‐linkages, with crucial preservation of sequence information (Figure 1).

**Figure 1:**
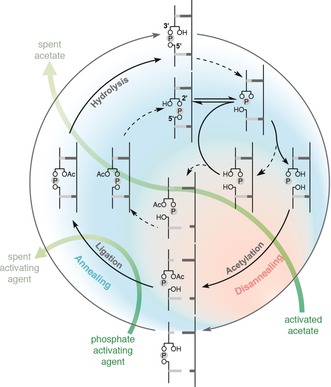
Proposed model for the recycling of backbone‐heterogeneous ribonucleic acids. Favored (bold lines) and disfavored (dashed lines) pathways resulting in the conversion of 2′,5′‐bonds into 3′,5′‐bonds are shown.

The initial 2′,5′‐cleavage results in a 2′,3′‐cyclic phosphate terminated oligomer, and hydrolysis of this cyclic phosphate is needed to access the terminal monophosphates involved in the acetylation and ligation steps of the synthetic stage. Cyclic phosphate hydrolysis is known to favor the 3′‐monophosphate product,^20^ and our chemoselective acetylation ensures that this isomer will be preferentially ligated, while fragments ending with a 2′‐phosphate will instead be recyclized. Importantly, we foresaw that since there was selectivity in favor of 3′,5′‐linkages in both the hydrolytic and synthetic stages, iterative cycling would progressively increase the ratio of 3′,5′‐linkages to 2′,5′‐linkages. Repeated acetylation, ligation, and hydrolysis would result in the turnover of large amounts of the acetylating and phosphate activating agents to spent products, but the energy thereby dissipated would “pay” the cost associated with enhanced selectivity of product formation.

To test this hypothesis, we first investigated the conditions required to selectively cleave an internal 2′,5′‐bond of the 13‐mer oligonucleotide **1** annealed to its complementary template **2**, and to further hydrolyze the terminal cyclic phosphate **3** generated by the cleavage (Figure 2 a). The reactions were performed in carbonate buffer (250 mm, pH 9.25, 21 °C) in the presence of different Mg^2+^ concentrations (0–40 mm), and their progress was monitored by HPLC (Figure 2 b, and Figure S1a,b in the Supporting Information).

**Figure 2:**
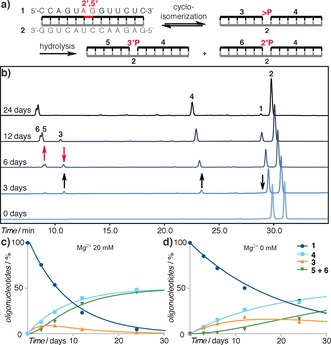
Hydrolysis studies. a) Schematic representation of the hydrolysis reaction: **1**=13 nt oligonucleotide with a single 2′,5′‐bond; **2**=13 nt template; **3**=6 nt primer 2′,3′‐cyclic phosphate; **4**=7 nt ligator; **5**=6 nt primer 3′‐phosphate; **6**=6 nt primer 2′‐phosphate. b) Representative HPLC chromatogram showing the progression of the hydrolysis reaction over time (Mg^2+^ 20 mm). c) Plot of the concentration of oligonucleotide **1** and fragments produced from it by hydrolysis in the presence of Mg^2+^ (20 mm). d) As (c) but in the absence of Mg^2+^.

Under these conditions, both the intact oligomers and the produced fragments are expected to be mainly helical (as estimated by melting point measurements; Figure S2), thereby preventing any degradation of single‐stranded RNA.^13^ The HPLC was performed under denaturing conditions, however, to allow separation and quantitation of the various species. The hydrolysis smoothly progressed to the expected products (**3**–**6**, Figure 2 a,b), and when the metal‐ion catalyst was included in the buffer, the reaction reached completion after 24 days of incubation (Figure 2 c and Figure S1c,d). Importantly, magnesium‐ion catalysis was found to be essential for the efficient opening of the cyclic phosphate to the 3′‐ and 2′‐monophosphate products (**5** and **6**, Figure 2 c; see Figure S1c versus Figure 2 d), which disfavors the reformation of **1** from **3**+**4** by equilibration and thereby boosts the rate of 2′,5′‐linkage hydrolysis. In parallel, the equivalent all‐3′,5′‐linked duplex (**7** and **2**, Table S1) was stable under these conditions, with only 1 % of degradation after 24 days (Figure S1d,e).

In the next stage of our proposed recycling model, the fragments produced by hydrolysis would undergo regioselective 3′,5′‐ligation by means of sequential protection and activation chemistry (Figure 1). To verify that the presence of a complementary strand would not affect the chemoselectivity of acetylation and subsequent ligation, we conducted a series of reactions in which 2′‐ and 3′‐phosphate‐terminated primers could react with a labeled ligator, either independently or in mutual competition (Figure S3a). Mixtures of primer(s), ligator, and template were acetylated (*N*‐acetylimidazole, 30 °C, 1 h) and then subjected to phosphate activation (*N*‐cyanoimidazole, Mn^2+^, 21 °C, 19 h). Mass spectrometry and gel analysis (Figure S3b–h) were in line with our previous observations and confirmed that preferential acetylation and ligation of 3′‐phosphate‐terminated primers took place in the presence of the template as they had in its absence.^8^

Finally, 2′‐O‐deacetylation of the newly synthesized oligonucleotide is required to access native RNA (Figure 1). In support of our model, the same conditions that promoted the cleavage of non‐natural bonds were found to effectively deacetylate RNA (Figure S4), thus completing the individual evaluation of the various stages of the recycling model.

Encouraged by these outcomes, we performed sequential rounds of hydrolysis and ligation with the aim of degrading and repairing the internal 2′,5′‐bond of oligomer **1**, thereby generating the fully 3′,5′‐linked product **7**. Initially, oligomers **1** and **2** were annealed and the resulting duplex was hydrolyzed in the presence of Mg^2+^ (250 mm carbonate buffer, 40 mm Mg^2+^, 21 °C). Aliquots of the reaction were taken at different time points (6, 12 and 24 days; Figure 3 and Figure S5a), desalted, subjected to acetylation–ligation and further hydrolyzed to promote deacetylation. After this first cycle, we were able detect the formation of a new peak previously absent in the mixtures (Figure 3 and Figure S5a), and spiking with **7** confirmed the formation of the fully 3′,5′‐linked product (Figure S5b). Moreover, during the last step, deacetylation and hydrolysis occur simultaneously; further cleavage of 2′,5′‐bonds takes place and the cyclic phosphate produced from the activation of **6**, or residual non‐acetylated **5**, is hydrolyzed back to these monophosphates and thus recycled. In accordance with the predictions of our proposed recycling model, subjecting the mixtures to further rounds of recycling resulted in the progressive enrichment in the corrected oligonucleotide **7** at the expense of **1** (Figure 3 and Figure 4).

**Figure 3:**
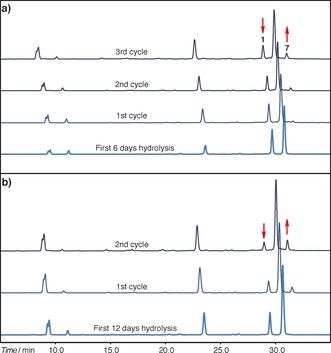
Recycling studies: HPLC chromatograms showing the recycling of **1** with formation of the fully 3′,5′‐linked product **7**. a) Three cycles of recycling with a 6 day hydrolysis stage. b) Two cycles of recycling with a 12 day hydrolysis stage.

**Figure 4:**
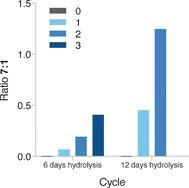
Enrichment of 3′,5′‐linkages at the expense of 2′,5′‐linkages. Plot of the **7**:**1** ratio versus the number of cycles of recycling (0–3, indicated by the color key) and comparison of cycles based on 6 days and 12 days of hydrolysis.

It is important to note that the degree of hydrolysis is critical in determining the **7**:**1** ratio after each cycle (Figure 4) and that extensive cyclic phosphate opening is required to shift the equilibration of **1** and **3**+**4** in favor of the cleavage, thus highlighting the central role of Mg^2+^ in the recycling scheme.

In this study, we have demonstrated the feasibility of non‐enzymatic energy‐dissipative conversion of 2′,5′‐bonds into 3′,5′‐bonds with retention of sequence. This supports a model in which repetitive cycles are likely to drive the evolution of backbone‐heterogeneous ribonucleic acids to the natural RNA used by extant biology. Fluctuations in temperature and salt concentrations, as could have occurred in open rock pores,^21^ might have created the appropriate geochemical settings to promote cleavage and repair. In this scenario, proofreading of 2′,5′‐bonds might have constituted the basis not only for sequence information recycling but also for the abiotic reshuffling and recombination of short oligomers,^22^ thereby enabling the system to progress along a trajectory that leads to functional RNA. From a wider perspective, our results suggest that other (pre)biological subsystems based on hydrolytically labile components might have been subject to optimization through energy‐dissipative recycling.^18^

## Conflict of interest

The authors declare no conflict of interest.

## Supporting information

As a service to our authors and readers, this journal provides supporting information supplied by the authors. Such materials are peer reviewed and may be re‐organized for online delivery, but are not copy‐edited or typeset. Technical support issues arising from supporting information (other than missing files) should be addressed to the authors.

Supplementary
