# Supplementary material for: Non‐Enzymatic RNA Backbone Proofreading through Energy‐Dissipative Recycling
Source: Angew Chem Int Ed Engl. 2017 May 3;56(23):6563–6. doi: 10.1002/anie.201703169 (PMC5488188; doi:10.1002/anie.201703169)
Supplement: Supplementary file 1 — Supplementary [file ANIE-56-6563-s001.pdf]

Angewandte Chemie
Eine Zeitschrift der Gesellschaft Deutscher Chemiker

Supporting Information

Non-Enzymatic RNA Backbone Proofreading through Energy-Dissipative Recycling

*Angelica Mariani and John D. Sutherland**

anie_201703169_sm_miscellaneous_information.pdf

Supporting Information

WILEY-VCH

Table of Contents

1. **Materials and Methods** **2**
2. **Experimental Procedures** **3**
3. **Supplementary Figures and Tables** **4**

1. Materials and Methods

Materials. Reagents and solvents were obtained from *Acros Organics*, *Ambion*, *Fisher Scientific*, *Severn Biotech Ltd.*, *Santa Cruz Biotechnology*, *Sigma-Aldrich*, or *VWR*. *N*-cyanoimidazole was purified by trituration with chloroform, filtration and evaporation of the solvent under a flow of argon before use. All the reactions were performed in nuclease-free water, not DEPC treated (*Ambion*). Ion-exchange resin was purchased in the Na^+ form (Dowex®50WX2, *Fluka*), pre-washed with 1 M NaOH aq. and then with water until the washings were neutral.

High-pressure liquid chromatography. *Preparative:* Varian PrepStar (*Agilent*). SAX-HPLC: DNAPac PA-100, 22 × 250 mm (flow rate 15 mL/min; detection 260 nm; solvents: A, 10 mM sodium phosphate buffer pH 11.5 - B, 1 M NaCl in A). *Analytical:* Dionex Ultimate 3000 (*Thermo Scientific*). RP-HPLC: AdvanceBio oligonucleotides, 4.6 × 150 mm analytical column (flow rate 0.5 mL/min; detection 260 nm; solvents: A, 400 mM hexafluoroisopropanol, 15 mM TEA pH 7.9 - B, 50 % A, 50 % MeOH). SAX-HPLC: DNAPac PA-100, 4 × 250 mm analytical column, with a DNAPac PA-200, 50 × 4 mm guard column (flow rate 1 mL/min; detection 260 nm; solvents: A, 10 mM sodium phosphate buffer pH 11.5 - B, 1 M NaCl in A). In Figure 2 and Figure S1, the %age of the various species at each time point was estimated by division of the integral of the HPLC peak at 260 nm for that/those species, by the sum of the integrals of the peaks for **1** and all the fragments derived therefrom. In Figure 4, the %age of the products was estimated by peak integration of the HPLC traces at 260 nm and by comparison of the relative %age areas after each cycle. Data analysis was performed using GraphPAD Prism (version 7.0b).

MALDI-TOF mass spectrometry. Voyager-DE Pro (*Applied Biosystems*). Matrix: 50 mg/mL ammonium citrate, 50 mg/mL 2,4,6-trihydroxyacetophenone in 50 % (v/v) acetonitrile in water (typically 2.0 µL of matrix were mixed with 0.5 µL of the sample mixture). Spectra were recorded in linear positive ionization mode, using a minimum of 300 shots/spectrum and calibrating to internal RNA standards. Data analysis was performed using mMass (version 5.5.0).

Gel electrophoresis. 20 % polyacrylamide, 8 M urea gels (0.75 mm thick, 20 cm long) were typically run at 27 W in TBE buffer. Fluorescence imaging was performed using Typhoon Trio variable mode imager (*GE healthcare*) and quantified using ImageQuant TL software (version 7.0). Gel staining with SYBR® Gold was additionally performed after quantification to reveal unlabeled oligonucleotides (10 min of incubation), and then imaged as described above.

Oligonucleotides. The 13 nt product 2′,5′-linked (Table S1, entry A) was purchased from *Gene Link* and additionally purified by preparative SAX-HPLC (gradient: 40-80 % B for 30 min). The target fractions were collected into an equal volume of 1 M TEAA pH 7 and then desalted using Sep-Pak®plus (*Waters*) C18 cartridges (cartridges were prewashed with water (10 mL) then MeOH (10 mL) before sample loading; desalting was performed by washing with 50 mM TEAA pH 7 (3 mL) before sample elution with 30 % (v/v) MeOH in 50 mM TEAA pH 7 aqueous buffer (5 mL)). The eluted fraction was lyophilized, redissolved in water and further desalted by RP-HPLC (gradient: 30-50 % B for 4 min and then 50-80 % B for 10 min). After lyophilization, the oligonucleotide was converted in the Na^+ form using pre-washed Dowex resin (mixing for 15 min before the resin was removed by filtration). RNA standards (Table S1, entries B-G and K) were purchased in HPLC-purified Na^+ forms from *Integrated DNA Technologies*. Additional RNA oligomers (Table S1, entries H-J) were synthesized as previously reported by us.[1] Oligonucleotide concentrations were determined by UV absorbance at 260 nm using NanoDrop® ND-1000 spectrophotometer.

UV melting measurements. UV thermal melting curves were acquired using a Varian Cary 6000i UV-Vis-NIR spectrophotometer equipped with a multi-sample Peltier temperature controller. All measurements were carried out in 10 mM Na_2HPO_4, 0.5 mM Na_2EDTA buffer (pH 7) and 100 mM NaCl, using 2.5 µM of each complementary strand. Prior to UV measurements, samples were degassed by heating at 95 °C for 4 min followed by brief sonication. Measurements were made in masked quartz cuvettes with a mineral oil layer over the sample to reduce evaporation. Absorbance versus temperature spectra were measured within a range of 10 – 90 °C, at 260 nm. The temperature was ramped at a rate of 0.5 °C/min, with absorbance measurements taken at 0.5 °C intervals. The oligonucleotides were annealed and equilibrated by the first heat-cool cycle, holding for 5 min at the maximum temperature. UV melting heat-cool runs were then conducted in triplicate holding for 2 min between ramps. Data analysis was performed using GraphPAD Prism (version 7.0b) and Microsoft Excel (Mac 2011) as previously described.[2] T_m were determined from the average of the three heating cycles.

2. Experimental Procedures

General procedure for desalting by ethanol precipitation. Oligonucleotides were desalted by addition of a 2 M imidazole nitrate buffer solution (pH 6.2, 1/10 volume), followed by a 3 M sodium acetate solution (pH 5.2, 1/10 volume) and absolute ethanol (to a final concentration of 75 % (v/v)). The resulting mixtures were typically kept at -20 °C for 2-4 h and then centrifuged for 30 min at 16000 × *g*. The supernatant was removed and the pellets were washed with 75 % (v/v) aqueous ethanol, before additional centrifugation (16000 × *g*, 10-30 min). The residual pellets were air dried before being redissolved in water.

General procedure for the hydrolysis reactions. Oligonucleotide **1** (or **7**) (80 µM) and its complementary template **2** (80 µM) were mixed in 200 mM NaCl (10.0 µL) and incubated at 95 °C for 4 min. The mixture was allowed to slowly cool to 21 °C before addition of 5.0 µL of a 1 M sodium carbonate buffer solution (pH 9.25) and either 5.0 µL of water or 5.0 µL of a 80 or 160 mM $MgCl_2$ solution (final concentrations: 40 µM each strand, 100 mM NaCl, 250 mM sodium carbonate, 0, 20 or 40 mM $MgCl_2$). The reaction was kept at 21 °C for 24 days and its progress was monitored after 3, 6, 12 (or 13) and 24 days by analytical SAX-HPLC (gradient: 30-80 % B for 35 min and then 100 % B for 5 min; the aliquots were desalted by ethanol precipitation before HPLC injection).

Procedure to assess the selectivity of the acetylation-ligation reaction of 3′p vs 2′p termini in a helical environment. The reaction was performed using a modified version of the procedure previously reported by us.[1] A mixture (6.0 µL) containing 33.3 µM of template (**2**), 33.3 µM of FAM-labeled ligator (**8**) and 33.3 µM of primer (**9**-**12**, or 16.7 µM each of 2′p and 3′p primer for competition experiments) was incubated at 70 °C for 4 min and then briefly cooled on ice. Acetylation was then conducted by addition of 1.0 µL of a 350 mM *N*-acetylimidazole solution (final concentration 50 mM) and incubation at 30 °C for 1 h. 0.5 µL of the acetylated mixture was kept for MALDI-TOF analysis and the remaining 6.5 µL were subjected to ligation in 200 mM imidazole nitrate buffer (pH 6.2), 10 mM $MnCl_2$ and 100 mM *N*-cyanoimidazole (final volume 9.3 µL). The reaction mixture was incubated at 21 °C for 19 h, then diluted with an equal volume of loading buffer (95 % (v/v) formamide, 5 % (v/v) glycerol, 50 mM EDTA, 0.5 mg/ml Orange G) and heated at 95 °C for 4 min before cooling briefly on ice. The reaction products were analyzed by gel electrophoresis, as described above.

Procedure to assess deacetylation under hydrolytic conditions. A mixture (10.0 µL) of 6 nt primer 3′p (**5**, 80 µM) and 50 mM of *N*-acetylimidazole was incubated at 21 °C for 5 h. A template ligation was then conducted using 20 µM of the acetylated primer, 20 µM of ligator (**4**) and 20 µM of template (**2**) in 200 mM imidazole nitrate buffer (pH 6.2), 10 mM $MnCl_2$ and 100 mM *N*-cyanoimidazole (final volume 30.0 µL). The reaction mixture was incubated at 21 °C for 19 h and then desalted by ethanol precipitation. The pellet was redissolved in 22.0 µL of water and divided into three aliquots. One fraction (2.5 µL) was directly analyzed by analytical SAX-HPLC (gradient: 30-80 % B for 35 min and then 100 % B for 5 min) and MALDI-TOF mass spectrometry. A second aliquot (9.5 µL) was mixed with 0.5 µL of a 4 M NaCl solution and incubated at 95 °C for 4 min. This mixture was allowed to slowly cool to 21 °C before addition of 5.0 µL of a 1 M sodium carbonate buffer solution (pH 9.25) and 5.0 µL of a 160 mM $MgCl_2$ solution (final concentrations: 100 mM NaCl, 250 mM sodium carbonate, 40 mM $MgCl_2$). The reaction was kept at 21 °C for 24 days and its progress was monitored after 6 and 24 days by HPLC and MALDI-TOF analysis (the aliquots were desalted by ethanol precipitation before use). The third fraction was used to perform a control reaction, employing the deacetylation procedure previously reported by us.[1] Thus the solution (10.0 µL) was lyophilized, redissolved in 20.0 µL of a 4.5 M NH_3 solution and heated at 40 °C for 1h before ethanol precipitation and MALDI-TOF/HPLC analysis.

Procedure for 2′,5′-linkages recycling. Oligonucleotide **1** (80 µM) and its complementary template **2** (80 µM) were mixed in 200 mM NaCl (75.0 µL) and incubated at 95 °C for 4 min. The mixture was allowed to slowly cool to 21 °C before addition of 37.5 µL of a 1 M sodium carbonate buffer solution (pH 9.25) and 37.5 µL of a 160 mM $MgCl_2$ solution (final concentrations: 40 µM each strand, 100 mM NaCl, 250 mM sodium carbonate, 40 mM $MgCl_2$). The reaction was kept at 21 °C and its progress was monitored by analytical SAX-HPLC (gradient: 30-80 % B for 35 min and then 100 % B for 5 min). Aliquots of 45.0 µL were taken after 6, 12 and 24 days of incubation, and desalted by ethanol precipitation. For each aliquot, the residual pellet was redissolved in 18.0 µL of water and the resultant solution was incubated at 70 °C for 4 min and then briefly cooled on ice. Acetylation was then conducted by addition of 3.0 µL of a 350 mM *N*-acetylimidazole solution (final concentration 50 mM) and incubation at 30 °C for 1 h. The mixture was then subjected to ligation in 200 mM imidazole nitrate buffer (pH 6.2), 10 mM $MnCl_2$ and 100 mM *N*-cyanoimidazole (final volume 29.3 µL), reacted at 21 °C for 19 h and then desalted by ethanol precipitation. The residual pellet was then redissolved in 17.25 µL of water and the resultant solution was divided into two aliquots. One fraction (14.25 µL) was mixed with 0.75 µL of a 4 M NaCl solution and incubated at 95 °C for 4 min. The mixture was allowed to slowly cool to 21 °C before addition of 7.5 µL of a 1 M sodium carbonate buffer solution (pH 9.25) and 7.5 µL of a 160 mM $MgCl_2$ solution (final concentrations: 100 mM NaCl, 250 mM sodium carbonate, 40 mM $MgCl_2$). The reaction was kept at 21 °C for the indicated days before HPLC analysis and a new round of reactions. The cycle was repeated for the indicated number of times, adjusting the volume of water to be added to the pellets, considering the reduction of available material after each cycle.

Procedure to estimate the yield of the templated acetylation-ligation, following pre-treatment with the hydrolysis buffer. The template (**2**, 20 µM), the ligator (**4**, 20 µM) and the FAM-labeled primer (**13**, 10 µM) were dissolved in the hydrolysis buffer (100 mM NaCl, 250 mM sodium carbonate buffer pH 9.25, 40 mM $MgCl_2$) and desalted by ethanol precipitation. The residual pellet was redissolved in 6.0 µL of water and the resultant solution was incubated at 70 °C for 4 min and then briefly cooled on ice. Acetylation

was then conducted by addition of 1.0 µL of a 350 mM *N*-acetylimidazole solution (final concentration 50 mM) and incubation at 30 °C for 1 h. The mixture was then subjected to ligation in 200 mM imidazole nitrate buffer (pH 6.2), 10 mM $MnCl_2$ and 100 mM *N*-cyanoimidazole (final volume 9.3 µL), reacted at 21 °C for 19 h and then diluted with an equal volume of loading buffer (95 % (v/v) formamide, 5 % (v/v) glycerol, 50 mM EDTA, 0.5 mg/ml Orange G). The resulting mixture was heated at 95 °C for 4 min, briefly cooled on ice and analyzed by gel electrophoresis, as described above. The reaction was performed in triplicate. Yield: 39 ± 2%.

3. Supplementary Figures and Tables

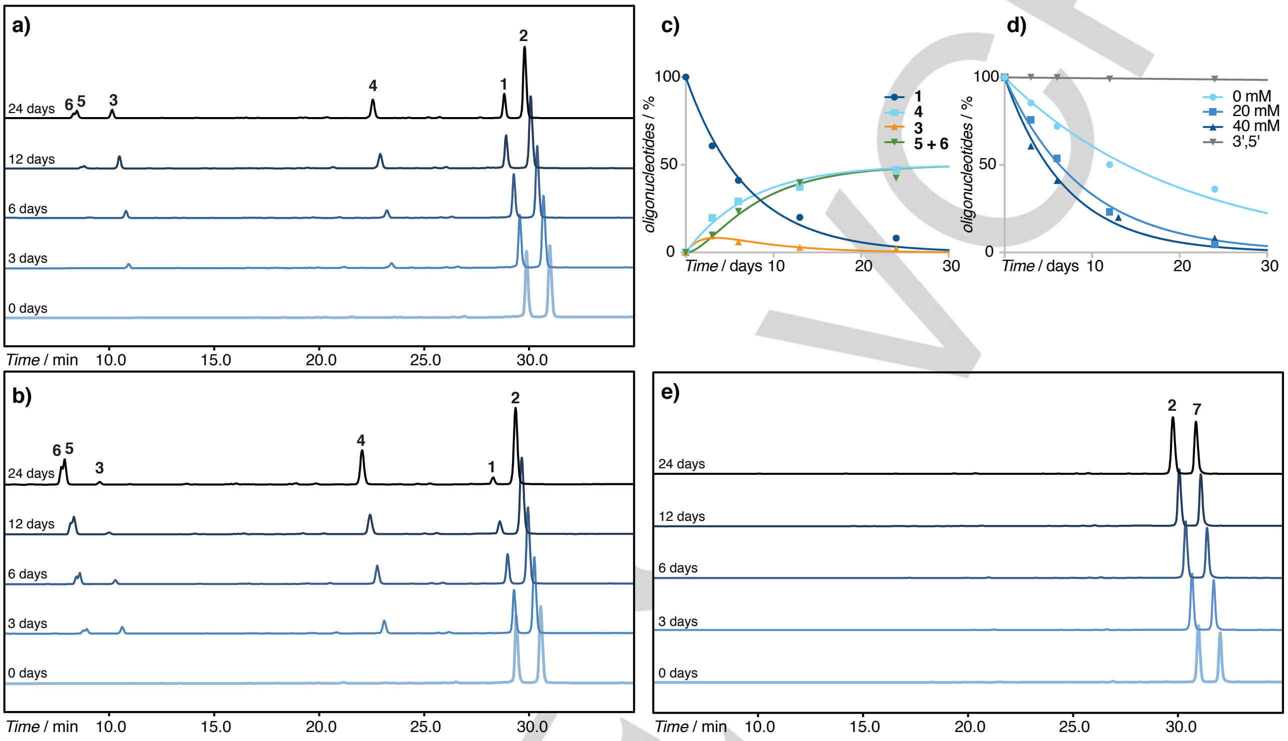

Figure S1. Hydrolysis studies. a) HPLC chromatograms showing the progression of the hydrolysis of **1** in a duplex formed with **2** over time (Mg^{2+} 0 mM). b) HPLC chromatograms showing the progression of the hydrolysis of **1** in a duplex formed with **2** over time (Mg^{2+} 40 mM). c) Plot of the concentration of oligonucleotide **1** and fragments produced from it by hydrolysis in the presence of Mg^{2+} (40 mM). d) Plot of the %age of **1** at different time points, performing the reaction in the presence of different Mg^{2+} concentration (0, 20, 40 mM) and comparison with the hydrolytic stability of the all 3′,5′-linked product **7** (Mg^{2+} 40 mM). e) HPLC chromatograms showing the lack of hydrolysis of **7** in a duplex formed with **2** over time (Mg^{2+} 40 mM).

WILEY-VCH

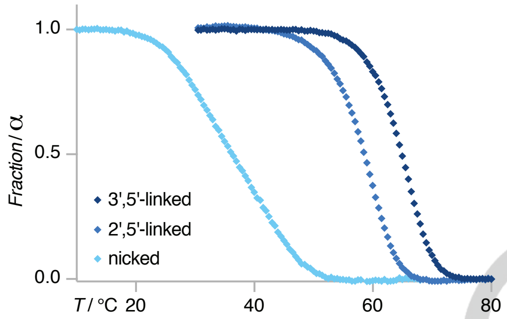

Figure S2. UV thermal melting curves for the all 3′,5′-linked duplex (**7** + **2**, T_m = 65.0 °C), duplex containing one 2′,5′-linkage (**1** + **2**, T_m = 58.0 °C) or duplex containing a nick (**4** + **5** + **2**, T_m = 35.3 °C). Experiments were conducted at 2.5 μM of each complementary strand in 10 mM Na_2HPO_4, 0.5 mM Na_2EDTA buffer (pH 7) and 100 mM NaCl. Mg^{2+} was omitted to avoid degradation. In the hydrolysis buffers at 21 °C (100 mM NaCl, 250 mM sodium carbonate buffer pH 9.25, 0-40 mM $MgCl_2$) the oligomers are therefore expected to be mainly duplex.

WILEY-VCH

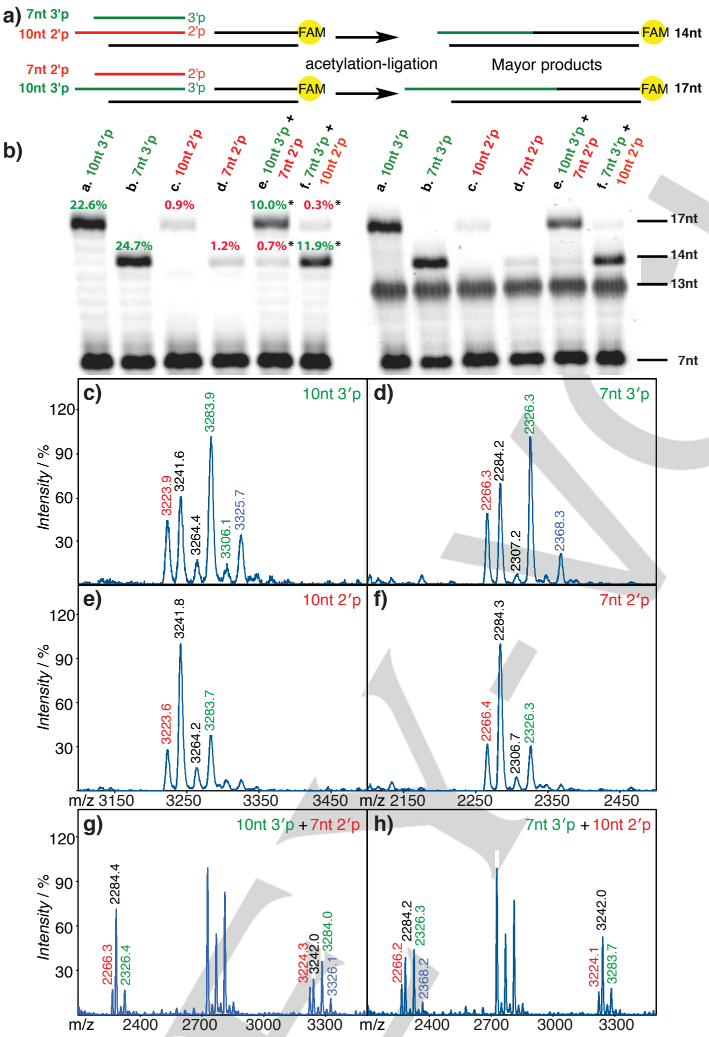

Figure S3. Templated acetylation-ligation. a) Schematic representation of the reaction: primers were either reacted independently or in competition. b) Gel electrophoresis analysis: primer(s) were included in the mixtures as indicated in each lane. Lanes **a**-**d**: assessment of the ligation selectivity for each independent primer; lanes **e**-**f**: assessment of the ligation selectivity in competition experiments. The products were quantified by fluorescence scanning (left, n.b. unligated primers and template not detectable) before SYBR Gold staining (right, n.b. primers could not be detected/distinguished from FAM-labeled ligator). Yields are referred to the total amount of labeled ligator. c-h) MALDI-TOF mass spectra of the mixtures after acetylation: the primer(s) present are as labeled in each spectrum. Peaks attributed to cyclic phosphate (numerical mass in red), unreacted primer (black), mono- (green) and bis-acetylated products (blue) and their adducts (see Table S2 for calculated masses). Unlabeled peaks in spectra g) and h) belong to the FAM-labeled ligator and its acetylated derivatives. *In competition experiments the concentration of each primer is halved compared to the independent reactions; as a consequence yields for lanes **e**-**f** are halved compared to lanes **a**-**d**, but the ratio 3′,5′:2′,5′ is maintained, thus indicating that selectivity is not affected by the presence of 2′p primers. The ligation yield of the 10 nt primer 3′p **9** (lane **a**) with the labeled ligator **8** was found to be lower than when performing the same reaction between the labeled 10 nt primer **13** and ligator **4** (yield: 45%). These differences maybe due to the effect of the FAM tag, which in **13** is attached to a 4 nt overhang, while in **8** is attached to the terminal nucleotide involved in the duplex and might therefore interfere with its correct annealing to the complementary template.

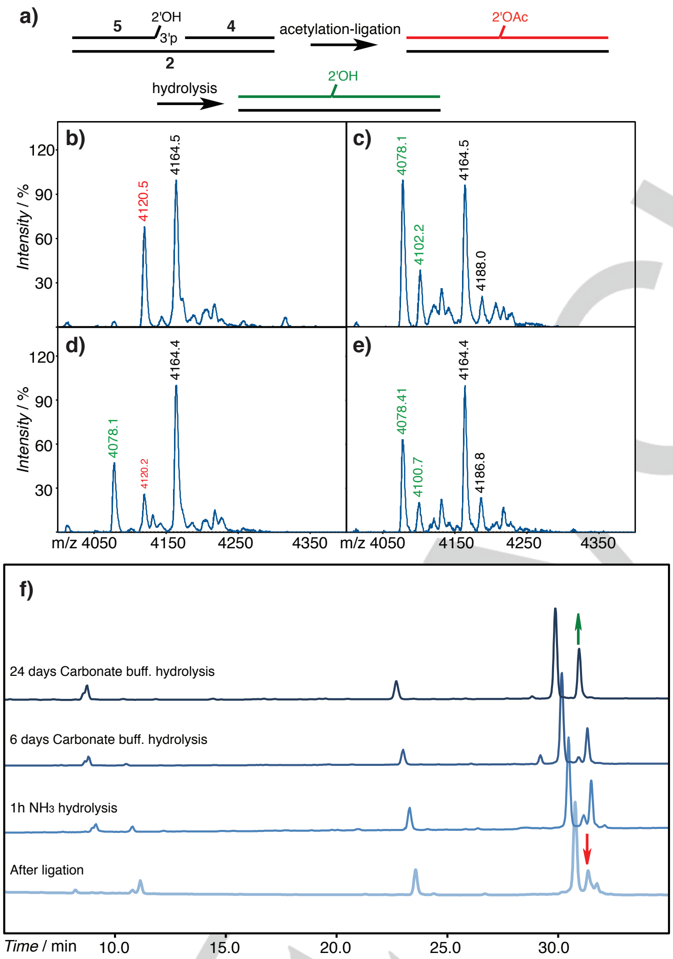

Figure S4. Deacetylation reaction. a) Schematic representation of the reaction. b) MALDI-TOF mass spectra of the mixture after ligation, c) after 1h of reaction with NH_3 (aq.), d) after 6 days of hydrolysis with carbonate/Mg^{2+} buffer, e) after 24 days of hydrolysis with carbonate/Mg^{2+} buffer. Peaks attributed to the 13 nt acetylated product (numerical mass in red), 13 nt deacetylated product (green), template (black) and their Na^+ adducts (see Table S2 for calculated masses). f) HPLC chromatograms of the mixtures showing the progress of deacetylation in different conditions (red arrow: acetylated product; green arrow: deacetylated product). The basic solvents (pH 11.5) used in SAX-HPLC caused partial deacetylation of the product during the running.

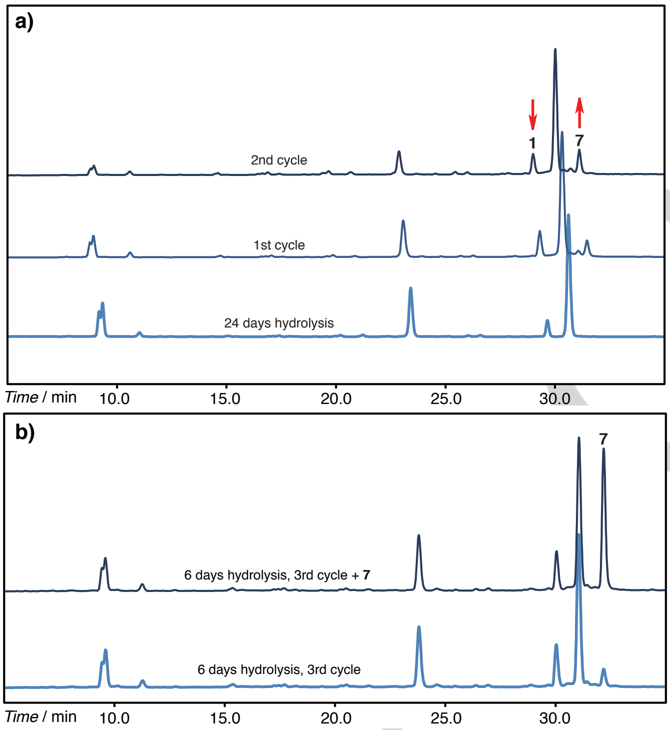

Figure S5. Recycling studies. a) HPLC chromatograms showing the recycling of 2′,5′-linkages: the hydrolysis reaction was stopped after 24 days of incubation and the mixture was subjected to two rounds of recycling, alternated by 6 days of hydrolysis. b) HPLC chromatograms confirming the identity of the new peak as the fully 3′,5′-linked product: 6 days of hydrolysis followed by three rounds of recycling, alternated by 6 days of hydrolysis (bottom) and the same sample spiked with **7** (top).

WILEY-VCH

Table S1. RNA oligonucleotides used in the present study.

Entry	Name	RNA sequence (5′-3′)
**A**	13 nt product 2′5′-linked (**1**)	CCAGUA-2′,5′-GGUUCUC
**B**	13 nt template (**2**)	GAGAACCUACUGG
**C**	7 nt ligator (**4**)	GGUUCUC
**D**	6 nt primer 3′p (**5**)	CCAGUA-3′p
**E**	13 nt product 3′5′-linked (**7**)	CCAGUAGGUUCUC
**F**	(6FAM)-7 nt ligator (**8**)	GGUUCUC-3′(6FAM)
**G**	10 nt primer 3′p (**9**)	UGUGCCAGUA-3′p
**H**	10 nt primer 2′p (**10**)	UGUGCCAGUA-2′p
**I**	7 nt primer 3′p (**11**)	GCCAGUA-3′p
**J**	7 nt primer 2′p (**12**)	GCCAGUA-2′p
**K**	(6FAM)-10 nt primer 3′p (**13**)	5′(6FAM)-UGUGCCAGUA-3′p

Table S2. Calculated masses of the products identified by MALDI-TOF mass spectrometry.

RNA species	Calculated Average Mass (Da)	
	**[M+H]$^{+}$**	**[M+Na]$^{+}$**
13 nt acetylated product	4120.5	4142.5
13 nt deacetylated product	4078.5	4100.5
Template	4164.6	4186.6
7 nt 2′,3′>p	2266.4	2288.4
7 nt 2′p/3′p	2284.4	2306.4
Mono-acetylated 7 nt 2′p/3′p	2326.4	2348.4
Bis-acetylated 7 nt 2′p/3′p	2368.4	2390.4
10 nt 2′,3′>p	3223.9	3245.9
10 nt 2′P/3′p	3241.9	3263.9
Mono-acetylated 10 nt 2′p/3′p	3283.9	3305.9
Bis-acetylated 10 nt 2′p/3′p	3325.9	3347.9

References

[1] F. R. Bowler, C. K. W. Chan, C. D. Duffy, B. Gerland, S. Islam, M. W. Powner, J. D. Sutherland, J. Xu, *Nat. Chem.* **2013**, *5*, 383.

[2] J. Xu, C. D. Duffy, C. K. W. Chan, J. D. Sutherland, *J. Org. Chem.* **2014**, *79*, 3311

Author Contributions

A.M. and J.D.S. designed the experiments and analyzed the data. A.M. performed the experiments and J.D.S. supervised the project. J.D.S. and A.M. co-wrote the paper.

WILEY-VCH
